# Cues and Attention in Parkinsonian Gait: Potential Mechanisms and Future Directions

**DOI:** 10.3389/fneur.2015.00255

**Published:** 2015-12-08

**Authors:** Daniel S. Peterson, Katrijn Smulders

**Affiliations:** ^1^Veterans Affairs Portland Health Care System, Portland, OR, USA; ^2^Department of Neurology, Oregon Health & Science UniversityPortland, OR, USA

**Keywords:** cues, dual tasking, walking, Parkinson’s disease, cognition

## Introduction

Gait in people with Parkinson’s disease (PD) is arrhythmic, small in amplitude, and variable (1–3). In addition, people with PD often exhibit reduced automaticity of movement (4), resulting in increased attention directed toward gait. This can be observed empirically when they have to perform a secondary task in addition to gait, so-called dual-task walking. In dual-task conditions, people with PD show larger impairments in gait than their healthy peers (5–7); for review, see Ref. (8). One strategy to improve gait in people with PD is cueing. Cueing is a well-established rehabilitation technique for improved locomotion in people with PD (9). In the clinic, auditory cueing is typically used to improve consistency and rhythmicity of steps. In individuals with PD who freeze, visual and auditory cues can also be used in a transient manner to break freezing events [for review, see Ref. (10)]. However, the mechanisms through which cueing improves gait are incompletely understood. The purpose of the current manuscript is to present proposed mechanisms of action of cueing. Further, we highlight the importance of cognition and, specifically, attention, in the efficacy of cueing. Finally, we present several possible directions for future research in the field.

Attention plays an important role in the efficacy of cueing. For example, as reduced movement automaticity may contribute to poorer gait function (e.g., smaller, more variable steps) in people with PD (4), external cues may act as pace-makers, taking the place of this additional cognitive control and reducing the amount of attention needed to maintain stable gait. This would mean that cued gait would allow more attention to be devoted to other secondary tasks, and one would expect lower dual-task costs (11). Alternatively, external cues may help to focus attention on gait. This should be particularly helpful in conditions that require more attention, such as walking while negotiating obstacles. If this were true, then one would expect to see a *prioritization* of the gait task over other tasks while using cues. Finally, it could be true that in specific circumstances and subgroups, external cues represent an additional cognitive task to walking, also requiring attention (12). Thus, cues may compete with gait for attentional resources and reduce gait quality during complex or attention-demanding environments.

Research has provided clues regarding the role of attention in cued gait. In a sub-analysis of the RESCUE trial (11), the effect of cueing was tested while completing either simple walking (no secondary task) or a complex secondary motor task – carrying a tray with glasses filled with water. Interestingly, results showed that gait speed improvements through cueing were only apparent while completing the complex motor task; a detrimental effect was observed during simple walking. In other words, the cue prevented gait slowing even while carrying the tray. These results suggest that cueing improves dual-task ability, and seem to support the idea that cues reduce attentional demands, thus freeing up attentional resources to secondary tasks. However, while this conclusion is plausible, it is also possible that cueing forced allocation of attention toward gait, potentially to the detriment of the secondary motor task performance. To distinguish between these two explanations, it would be necessary to capture the performance on the secondary task (i.e., what happened to the glasses on the tray) in the complex gait plus cueing condition. If cueing reduces the required amount of attention for gait, we would expect the cues not to impact performance carrying the tray. In contrast, if cueing led to allocation of attention specific to gait (prioritization of gait over secondary task), then deterioration on the tray-carrying task would be expected.

In a more recent study (13), a secondary cognitive task was used to evaluate the effects of attentional or “internal” cueing (i.e., “think about taking larger steps”), and “external” auditory cueing during attention-demanding situations. Results showed that attentional cueing, but not auditory cueing, resulted in improved gait velocity, possibly due to the specific focus on length of steps in that condition. Interestingly, the benefits of attentional cueing were retained during dual tasking. Although not formally analyzed, the authors also reported no differences in the cognitive task during the different conditions. This lends some support to the idea that cueing, in particular, “internal” attentional cueing, may reduce the amount of attention needed for gait. Additional indirect evidence for this hypothesis comes from a study where PD subjects had to respond to auditory stimuli that were presented regularly, while walking. A concern in this dual-task study was that presentation of these secondary-task stimuli at a fixed interval (1 or 2 s) could act as external cues, thereby improving gait. To test this, an additional condition was added in which auditory stimuli were presented at random intervals (1–3 s). Interestingly, the more difficult 1s condition (higher time pressure) yielded less dual-task costs on gait than the 2 s or variable interval conditions, possibly reflecting a cueing effect. However, during this 1 s condition, we also observed higher dual-task costs on the auditory cognitive task (i.e., slower and less accurate responses). Thus, although the cognitive stimuli may have worked as a cue to help to keep gait speed up, this came at a price, namely, drawing attention from the cognitive task. Given these partially conflicting results, additional work directly assessing performance on both gait and cognitive tasks with and without cues and dual tasks will be necessary to elucidate these interactions.

The concern that external cues might be detrimental for complex gait due to attentional costs to attend to the cues is not supported in the abovementioned studies. This concern was also directly addressed in a study looking at obstacle avoidance performance while walking on a treadmill (12). This challenging task has previously been shown to demand considerable attention (14–16). In line with previously mentioned studies, this complex motor task was not affected by external cues, providing further evidence that external cues do not add additional attentional costs during walking, thus worsening gait performance.

Together, these findings confirm that cueing improves gait even in complex or cognitively challenging environments. However, one should note that not all gait parameters change in a similar fashion through cueing. For example, spatial parameters such as gait speed and stride length are typically improved during visual, attentional, and rhythmic cues, while temporal cues such as cadence are unaffected (11, 17, 18). These results are not fully consistent however, as Lebold and Almeida recently showed that visual cues increased stride length, but reduced cadence in people with PD (19). Further, recent work has begun to investigate the effects of cues on gait stability parameters, such as spatiotemporal variability. However, there is currently a paucity in high-quality research on the effect of cues on these stability metrics that may be more reflective of quality of gait.

## Role of Cognitive Impairments

A deeper understanding of shifts in attentional load and prioritization during cued gait is particularly important for individuals with limited cognitive resources. This population often exhibits more pronounced gait and postural decline (20–22), including freezing of gait (FoG) (23), and evidence suggests that these deficits are at least partially due to limited attentional capacity (22). Indeed, cognitively impaired elderly exhibit larger dual-task costs on gait than their cognitively intact peers (24–26).

However, previous research on cued gait in PD has been focused primarily on people who were cognitively intact. To effectively use cues, one must have sufficient cognitive capacity to (1) not be overwhelmed by multiple, perhaps distracting stimuli, and (2) be able to deploy the cues at the necessary moment. Thus, people with cognitive dysfunction may have difficulty utilizing cues. This specific concern was addressed in a study evaluating cueing effects in PD with mild-to-moderate cognitive impairment (MMSE 15–26) and found that cueing was feasible and effective (27). However, the performance on the secondary task was not reported, raising the possibility (as noted above) that cueing may have shifted prioritization to the gait task, resulting in poorer performance on the secondary gait task. Interestingly, Willems and colleagues (28) showed that PD patients who freeze benefited from cueing only when the frequency of the cues matched their step frequency, whereas non-freezers also benefited from all cue presentations. Further, PD patients who freeze have been shown to exhibit a dramatic performance loss while dual-tasking despite the presence of cues. This was not the case in PD without FoG (29). These findings could be attributed to the poorer executive and attentional functions in PD with FoG (30–34). However, it cannot be ruled out that other, motor-related differences between PD patients with and without FoG underlie this lack of cue-efficacy in people who freeze. Given these results, additional work administering cues with and without secondary task in individuals with more severe cognitive difficulty is warranted.

## Future Directions

Given current evidence regarding gait and attentional impairments in people with PD, certain considerations could be made to improve the efficacy of cues in the face of secondary cognitive challenges. Several possible approaches are outlined below.

First, different cue modalities can affect different gait characteristics. Rhythmical auditory cues were shown to be more effective than rhythmic visual or proprioceptive cues to improve gait speed, possibly due to a higher degree of integration of auditory rhythmical cues into stepping (27). Alternatively, visual cues including projecting lines on the ground have previously been shown to be more effective than auditory cues at improving step length (35, 36); however, a recent meta-analysis reported that visual cues did not result in additional improvement in stride length over auditory cues (37). Proprioceptive cues may also be beneficial for people with PD. Recent studies have suggested that walking on a treadmill improves gait in people with PD (38), and these improvements are maintained during subsequent over ground walking (39). Although the mechanism of gait improvements during treadmill walking is not fully understood, proprioceptive cues from the treadmill may contribute to these improvements. Such proprioceptive cues require little to no cognitive processing or attention (40, 41), and therefore may be particularly beneficial for individuals with cognitive challenges. However, there is a relative lack of literature investigating proprioceptive cuing, and additional work will be necessary to fully understand the effect of this type of cueing on gait in people with PD. In particular, treadmill locomotion may be different than overground walking in a number of ways (42, 43), including, but not limited to proprioceptive cues. Therefore, studies investigating the specific effects of proprioceptive cues, controlling for other treadmill related sensory inputs, are warranted.

A final perspective on cue modality choice is the personal preference. For rhythmical cues, auditory cueing seem to be preferred over other modalities. Nieuwboer and colleagues showed that when given the choice of three different rhythmic cues, proprioceptive (vibrations on the wrist), visual, and auditory cues, 67% of participants chose auditory, 33% chose proprioceptive, and 0% chose visual (44). However, no studies have compared personal preference of spatial (e.g., lines on the ground) to rhythmic cues.

Another aspect of cueing is whether to limit it to training circumstances and hope for transfer to situations without cues or to broaden its use to everyday life. Extending the use of cues outside training situations, like walking over a busy sidewalk, will introduce a number of challenges, but may be necessary given the relatively limited success of cue training over longer follow-up periods (44). Technological advances may help to reduce these challenges. For example, cues that are automatically initiated by arrhythmic gait (captured by body-worn sensors) may represent an important tool for cue utilization (45). Instead of continuous cueing, this “just in time” or “on demand” cueing might be more practical to use during daily activities. In addition, automatically initiated cueing could be helpful for individuals with cognitive dysfunction who may not be able to initiate cueing at the appropriate moment. Incorporation of visual cues which are integrated in the environment, so-called “augmented reality cueing” is also being developed. For example, Espay and colleagues designed a system that projects a tiled floor pattern over the real environment, with the optical flow of the tiles adapted to the individual’s walking speed (46). Another example is the use of Google glass to present visual optical flow as cues (47). While these forms of cueing are promising, additional testing will be necessary to identify which approaches are beneficial for patients.

## Conclusion

Cues can improve gait in people with PD. Different hypotheses have been put forward to explain these improvements, but further research is necessary to understand how cues improve gait. In particular, capturing changes in performance in both gait and cognitive tasks during cued dual-task will help elucidate underlying mechanisms. These interactions are particularly important to understand how individuals with reduced cognitive capacity utilize cues in distracting environments. Further, recent research has provided insight into how and when to choose cue modality, as well as how technology can integrate cues into real world environments, further reducing structural or cognitive interference. Continued investigation of these topics will improve our ability to utilize cues to improve gait in people with PD.

## Author Contributions

Both authors (DP, KS) contributed equally to the drafting and revision of this manuscript.

## Conflict of Interest Statement

The authors declare that the research was conducted in the absence of any commercial or financial relationships that could be construed as a potential conflict of interest.
